# Composite Central Face Design—An Approach to Achieve Efficient Alginate Microcarriers

**DOI:** 10.3390/polym11121949

**Published:** 2019-11-27

**Authors:** J.F.A. Valente, J.R. Dias, A. Sousa, N. Alves

**Affiliations:** 1 CDRsp-IPL-Centre Rapid and Sustainable Product Development, Polytechnic Institute of Leiria, 2430-028 Marinha Grande, Portugalnuno.alves@ipleiria.pt (N.A.); 2 CICS-UBI-Health Sciences Research Centre, Universidade da Beira Interior, Avenida Infante D. Henrique, 6200-506 Covilhã, Portugal; angela@fcsaude.ubi.pt

**Keywords:** alginate, Design of Experiments, electrospraying, microparticles, protein release

## Abstract

Microparticulated drug delivery systems have been used as promising encapsulation systems for protecting drugs for *in vitro* and *in vivo* applications, enhancing its stability, providing an increased surface to volume ratio, reducing adverse effects, and hence an improvement in bioavailability. Among the studied microparticles, there is a rising interest in the research of alginate microparticles for pharmaceutical and biomedical fields confirming its potential to be used as an effective matrix for drug and cell delivery. Moreover, calcium alginate has been one of the most extensively forming microparticles in the presence of divalent cations providing prolonged drug release and suitable mucoadhesive properties. Regarding the above mentioned, in this research work, we intended to produce Ca-alginate micro-vehicles through electrospraying, presenting high encapsulation efficiency (EE%), reduced protein release across the time, reduced swelling effect, and high sphericity coefficient. To quickly achieve these characteristics and to perform an optimal combination among the percentage of alginate and CaCl_2_, design of Experiments was applied. The obtained model presented to be statistically significant (*p*-value < 0.05), with a coefficient of determination of 0.9207, 0.9197, 0.9499, and 0.9637 for each output (EE%, release, swelling, and sphericity, respectively). Moreover, the optimal point (4% of alginate and 6.6% of CaCl_2_) was successfully validated.

## 1. Introduction

Natural polymers such as alginate have been extensively used in the drug carrier’s production mainly because they are easily obtained, biocompatible, and inexpensive. Alginate polymers can be extracted from brown seaweeds and bacterium, being their compositions depending upon the source from which they are isolated. Therefore, the production of alginates with specific structures can also be achieved through enzymatic modification using mannuronan C-5 epimerases. They come from a family of unbranched binary copolymers of (1,4)-linked β-D-mannuronic acid (M) and α-l-guluronic acid (G) with widely varying compositions and sequential structures [1,2].

These biopolymers have the ability to produce microparticulate vehicles broadly used for cells, drugs and enzymes delivery [2]. Additionally, these beads have cheap, fast, and easy production procedures, which make them extremely suitable for the applications above reported. Furthermore, there are two different ionotropic gelation methods used to produce alginate microparticles (MPs): the extrusion dripping method and the solvent emulsification method, being the first related with external and the second with internal gelation, respectively. Until now, there are some published works related to the formulation parameters on alginate MPs [3]. However, most of them are focused on the influence of the release profile [4], crosslinker, and its ratio [5,6], on the combination of some particular parameters of the MPs produced (like size and sphericity [7]), or in the use multimaterial particles [8]. The mainly applied technique for the MPs production is the extrusion dripping and, during this process, the alginate is extruded through a capillary/nozzle and dropwise into a multivalent ion solution [7]. Calcium (Ca^2+^) is the most studied ion to be combined with alginate because it is clinically safe, easily accessible, and economically viable. The combination between Ca^2+^ and alginate is also a very well studied and defined process, where the divalent cation binds to two carboxyl groups on the adjacent molecules, which can be called the “egg-box” structure [6,9]. The use of extrusion to produce the MPs usually results in larger structures, however, to overtake this inconvenient, electrospraying has been applied. The methodology used is based on pumping a liquid into a nozzle to which the high voltage (kV range) is applied during electrospraying to form MPs [10]. Using electrospraying enables several advantages, such as the improvement of dissolution rate of poorly water-soluble drugs, batch-scalability, reproducibility, effective encapsulation, and simple setup configurations in MPs production [11]. Furthermore, the selection of the best conditions for the formulation of Ca-alginate micro-vehicles with the ideal characteristics by electrospraying is a difficult task, regarding the influence of several parameters that should be controlled and optimized. The search of the optimal condition for each parameter, by varying one parameter while all others are fixed, is time-consuming, requires a lot of experiments, and the interaction effects between parameters can be missed, which sometimes is essential for better optimization of a process. To overcome these drawbacks, the Design of Experiments (DoE) can be used, a statistical tool that enables a systematic and simultaneous combination of parameters, which can influence the Ca-alginate micro-vehicles, in a faster and cost-efficient way when compared with the common random experiment approach [12,13].

Taking this information into consideration, in this research work, DoE was applied to find out the most adequate formulation of Ca-alginate micro-vehicles obtained through electrospraying, in order to promote high encapsulation efficiency (EE%), reduced protein release across the time, reduced swelling ability, and a high sphericity coefficient. With a high EE% it is intended that a few amounts of particles had the ability to deliver large amounts of the target biomolecule. Furthermore, to produce a delivery vehicle able to promote a release as long as possible, it is also important to consider the swelling capability of the MPs. A large swelling phenomenon can lead to a disintegration of MPs and rapid release of the target molecule, which is not desirable in micro-vehicles formulation of sustained release applications [6]. Additionally, the MPs sphericity is also important to be controlled to obtain reproducible results in drug assays and also, because beads with a well-defined geometry allow precise and controllable drug release profile [14,15].

## 2. Materials and Methods 

### 2.1. Materials

Sodium alginate was purchased from Sigma Aldrich (St. Louis, MO, USA) and calcium chloride (CaCl_2_) and Tris(hydroxymethyl)aminomethane (Tris) were purchased from Sharlau (Barcelona, Spain). Moreover, BSA was purchased from GERBU (Heidelberg, Germany) and Thermo Scientific (Waltham, MA, USA) supplied Bicinchoninic acid (BCA) assay.

### 2.2. Methods

#### 2.2.1. Alginate Microparticles Production

Alginate solution was prepared by dissolving sodium alginate in distilled water and then BSA was added to the solution and was stirred until full protein/polymer dissolution. After, the solution was transferred to 10 mL syringe capped with a blunted stainless needle (22 G) and high voltage electric field (9 kV) was applied to the polymer solution fed, at a constant flow rate (1.5 mL/h) by using a syringe pump to draw alginate beads toward a beaker containing calcium chloride solution (as the gelation bath) placed on the grounded electro conductive plate, at 17 cm of the tip of the alginate syringe needle. The produced beads were cross-linked and hardened in a gelation bath (gelation solutions were used made of different divalent ions namely: CaCl_2_, BaCl_2_, CuCl_2_, and ZnCl_2_) for 30 min and was then washed in Tris-HCl pH 7.4. Then, the MPs were dried at room temperature using a micropipette to collect the entire gelation bath (no aggregation phenomena was visualized) before the beginning of protein release studies.

#### 2.2.2. Design of Experiments (DoE)

To optimize the alginate MPs production and maximize the EE%, and sphericity while minimizing the release and swelling, a composite central face design (CCF) design was applied. Concerning that, two factors (inputs) were taken into account, namely the % of alginate used during the electrospraying and the % of CaCl_2_ used in the cross-linked step for the MP production. The responses (outputs) considered for this DoE were the EE% and protein release after 24 h, the MP swelling capability and sphericity.

The inputs were studied at three levels (−1; 0; and +1) and the range was defined according to preliminary results obtained from the random experiments approach. From these initial experiments, it was settled the maximum (10% *w*/*v*) and minimum (1% *w*/*v*) amount of each compound (alginate and CaCl_2_) that was able to perform stable MPs or, according to the total solubility.

After to accomplish all the experiments suggested by the DoE and introduce the respective outputs, a statistical analysis was performed through Design Expert version 11 software (StateEase, Minneapolis, MN, U.S.A.). The generalized second-order polynomial model equation used in the response surface analysis is presented below (Equation (1)):

(1) $$Y\;=\;\beta_{0}\;+\;\beta_{1}\;X_{1}+\;\beta_{2}\;X_{2}\;\;+\;\beta_{11}\;X_{1}^{2}\;+\;\beta_{22}\;X_{2}^{2}\;+\;\beta_{12}\;X_{1}X_{2}.$$

#### 2.2.3. Encapsulation Efficiency

The MPs were also evaluated concerning its EE% ability using a BCA protein assay colorimetric method for protein detection and quantification. The BCA method is based on the reduction of Cu^2+^ to Cu^+^ by protein with the highly sensitive and selective colorimetric detection of the cuprous cation (Cu^+^). This assay leads to a purple-colored reaction product produced through the gelation of two molecules of BCA with one cuprous ion [16]. Regarding the above mentioned, the EE% was assessed in the gelation medium after MP formation. The BCA measurements were performed using a microplate reader (SPECTROstar Nano, BMG Labtech, Ortenberg, Germany) at 562 nm. A calibration curve with known concentrations of BSA was predetermined, and subsequently, the loading capacity was calculated by the Equation (2):

(2) $$\mathrm{EE}\%=\frac{Actual\;drug\;loading}{Theorical\;drug\;loading}\;\times 100.$$

#### 2.2.4. *In Vitro* Release Studies

The *in vitro* BSA release from MPs was tested in a Tris-HCl buffer solution (pH 7.4) being firstly the samples placed in falcons with 1 mL of Tris-HCl buffer and then placed on an incubation chamber at 37 °C. At certain time intervals, the supernatant was recovered and replaced by fresh Tris-HCl. The BSA concentration measurements were then performed using a BCA protein assay kit in triplicate. The amount of released protein from the samples was defined by Equation (3):

(3) $$Release\;\left(\%\right)=\frac{M_{t}}{M_{\infty }}\times 100,$$

where *M_t_* is the amount of released protein from the MPs at time t and *M∞* is the amount of protein pre-loaded in MPs.

#### 2.2.5. Swelling Behavior

The swelling properties of MPs were characterized in Tris-HCl buffer (pH 7.4). These carriers were placed in an eppendorf with 1 mL of swelling solution and allowed to swell at 37 °C. After 24 h the swollen MPs were weighed. The swelling ratio was evaluated by Equation (4):

(4) $$Swelling\;ratio\;\left(\%\right)=\frac{W_{t}-W_{0}}{W_{0}}\times 100,$$

where *Wt* is the final weight and W_0_ is the initial weight of MPs [17].

#### 2.2.6. Determination of the Sphericity Coefficient

To determine sphericity, images of at least 10 MPs of each run were taken (microscope micros Austria). The measure of the minimum and maximum diameter of the particles, as well as the area and perimeter of the MPs were performed using the software Microvisible. Then, the coefficient of sphericity of each bead was given by Equation (5):

(5) $$sc=\frac{4\pi A}{p^{2}},$$

where *A* is the projection surface area and p is the perimeter obtained by the image analysis. Beads with an SC near to 1 are considered spherical [18].

## 3. Results

### 3.1. Design of Experiments

An empirical model able to predict the optimal mathematical combination of alginate and CaCl_2_ using a CCF and a response surface methodology (RSM) was performed. This particular design was chosen mainly because it is more inclusive and could not consider points outside of the ranges established for the inputs, for which it is expected unsatisfactory results [19]. It is also important to refer that some preliminary studies were performed to establish the range of concentrations applied in the Ca-Alginate MPs production. Considering this initial screening, it was defined to use CaCl_2_ between 1% and 10% and, in the case of alginate polymer, concentrations between 1% and 4% were applied. Moreover, other parameters involved in the MPs production by electrospraying were fixed, according to some literature data, to be possible evaluate the precise importance of the Ca^2+^ and alginate in the MPs production. Among these parameters were: voltage applied (9 kV), distance between the nozzle tip and the surface of the crosslinking solution (17 cm), and needle size (22 G). These chosen parameters were performed in accordance with a previous work published by Partovinia and co-workers (2019) [7] in combination with our experience handling our electrospraying apparatus.

Considering the defined inputs, the Design Expert software suggested to perform eleven experiments and the respective outputs were recorded in supporting information. Three central points, highlighted at grey (Table in S1) were tested under the same conditions in order to access the model reproducibility. The multiple regression Equations (6)–(9) resultant from the Design of Expert 11.0 provides the influence and interaction that the chosen inputs (% alginate and % CaCl_2_) have on the outputs, namely the EE% and release after 24 h, swelling and also the MPs sphericity.

**EE% =** + 74.3 + 9.95 × A − 4.83 × B + 2.48 × AB + 0.25 × A^2^ − 8.10 × B^2^. (6)

**Release =** + 65.56 − 13.78 × A − 0.00 × B + 2.83 × AB + 5.19 × A^2^ + 9.44 × B^2^. (7)

**Swelling =** + 25.95 − 0.00 × A − 5.17 × B − 9.5 × AB − 29.37 × A^2^ − 7.87 × B^2^. (8)

**Sphericity =** + 0.98 + 0.053 × A + 5.00E − 003 × B − 2.50E − 003 × AB − 0.056 × A^2^ − 0.021 × B^2^. (9)

**Figure 1:**
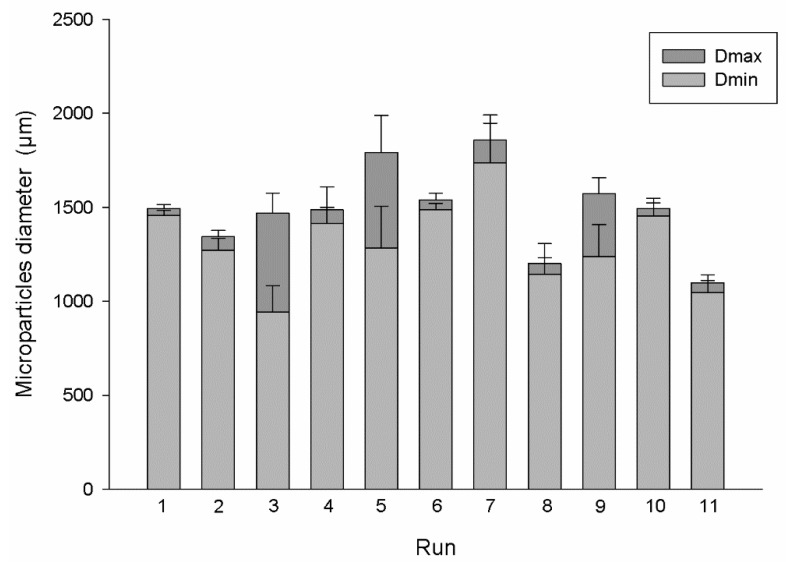
Maximum and minimum particles diameter obtained using the conditions suggested in each run by the Design of Experts (*n* = 10).

Through the obtained results, it is possible to observe that increasing the % of alginate leads to a positive effect on the EE% and on the MPs sphericity and a negative effect on the protein release. This behavior could be justified by the tighter and stiffer alginate mesh produced when high polymer concentrations were applied, which will capture the protein inside the MPs and will promote the formulation of spherical beads with a uniform structure [20]. However, and as stated through the obtained results, this tightening of the alginate mesh will decrease the protein release as it will have more resistant to pass through the alginate, taking more time to be released (which is favorable regarding the main goal of this research work). Therefore, there are already some previous studies referring that low amounts of alginate could lead to bead deformation during the collision with the gelling solution [21]. However, these authors, also conclude that a large increase of the alginate concentration (5–8%) lead MPs to have a pear-shaped structure. Being the sphericity intrinsically related with the particles size is possible to predict the degree of sphericity through the maximum a minimum diameter of the MPs. Concerning this, Figure 1 shows the sizes presented by the particles produced during the different runs suggested by the Design of Experts.

Besides, an increase in the % of Ca^2+^ seemed to favor sphericity and disfavor the swelling, probably because it intensifies the alginate crosslinking ability. This effect will improve the organization of the molecules and the stability/homogeneity of the MPs structure, thus avoiding water ingress and consequent swelling and disintegration of the MPs. Additionally, the increase of Ca^2+^ ions leads to an increase of the gelation kinetics accelerating the drug entrapment and the beads stabilization [22].

Furthermore, it is interesting to observe that from the interaction between “Alginate × CaCl_2_” (although for the output EE%, the alginate and the CaCl_2_ presented different behaviors when analyzed alone) the alginate behavior out-stood its influence over the CaCl_2_. From the interaction of both inputs it was also possible to see that the increase of both the polymer and reticulation agent led to a negative effect of the beads sphericity, which could be related with a phenomenon presented by Partovinia and collaborators (2019) [7] where the best sphericity results are achieved when a middle term condition of alginate/CaCl_2_ is used.

### 3.2. Three-Dimensional Response Surface Plots

**Figure 2:**
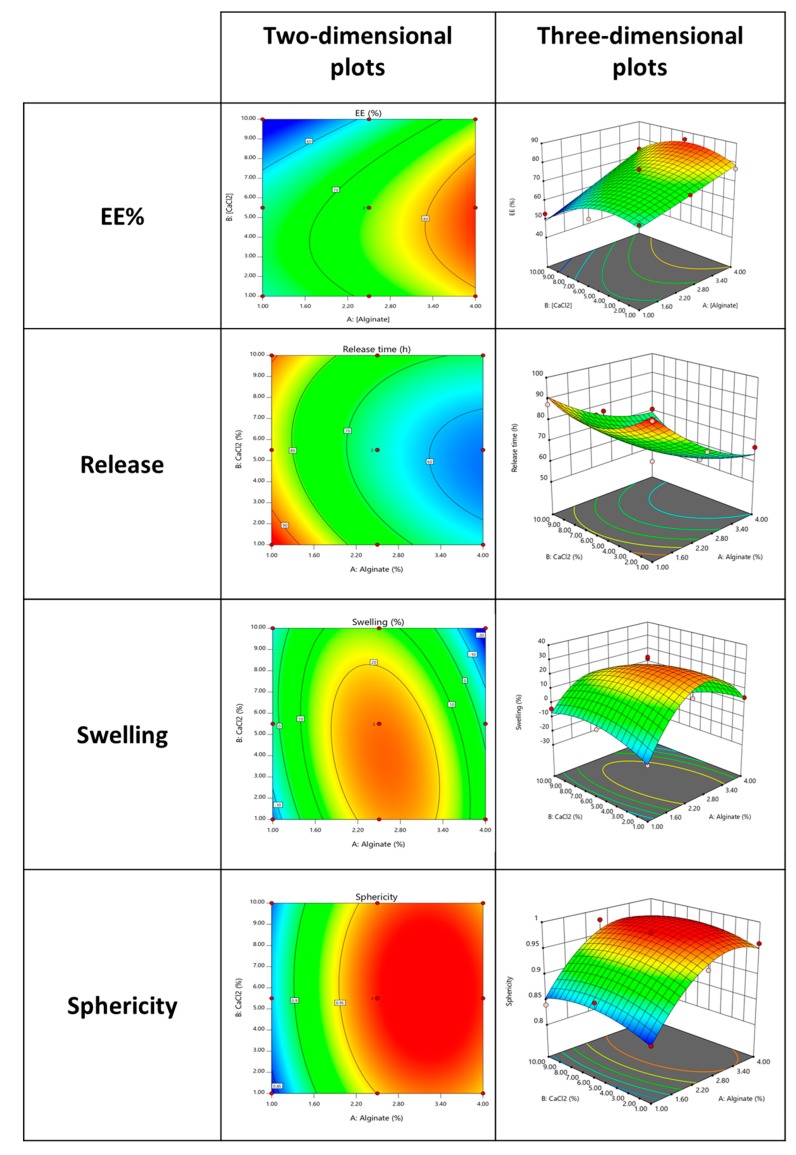
Contour and three-dimensional response surface plot of the interaction of the different variables (% of alginate and % of CaCl_2_) and its effect on EE%, release, swelling and sphericity.

After the analysis of variance (ANOVA) was performed with multiple regression analysis, three- and two-dimensional response surface plots were generated based on the reduced polynomial equations, which contained only the statistically significant terms to visualize the manipulating impact of the variables on the responses [23]. Additionally, the different colors intensity present on the plots present in Figure 2 represents the range for optimal points. Accordingly, blue represents the lowest response followed by green, yellow, and finally red, which represents the largest interaction. The ellipticity obtained in the plots also indicates the order of the interaction that occurs between the chosen variables.

From the obtained results for the output EE%, a large influence of high amounts of alginate when combined with the middle concentration of the CaCl_2_ was visible. The literature has already demonstrated that an increase in crosslink density will reduce the free volume spaces within the polymer matrix and further will promote a reduction of the EE% [24]. Furthermore, through this analysis was notorious that if extremely high or low amounts of CaCl_2_ were applied, a reverse behavior on the release was obtained (as observed by the negative effected presented by the CaCl_2_ on the output protein release).

Relating these plots with the ones obtained for the release it is interesting to observe that they appeared to be exactly the inverse, which means that the release increased with the application of low amounts of alginate and also middle concentrations of CaCl_2_ negatively influenced this output.

Moreover, swelling and sphericity have similar behaviors with the variation of the inputs being however the swelling more influenced by the middle concentrations of the alginate and CaCl_2_. In case of sphericity, after the achievement of a certain concentration of alginate, this response is equally influenced regardless of the amount of CaCl_2_ applied. Moreover, previous results provided by the multiple regression equations were the interaction between “Alginate × CaCl_2_” was explored and demonstrated to present a negative effect on sphericity.

Generally, these results suggest the inputs were well chosen and have a fundamental role in the MPs formulation, namely in its swelling and sphericity, and in the EE% and release phenomenon.

### 3.3. Statistical Analysis of the DoE Models

The analysis of variance (ANOVA) was performed to evaluate the significance of the used model (Table 1 and Table S2). From this analysis several important statistical parameters were accessed namely, the coefficient of determination (R^2^) that is responsible for showing if data points fit the statistical model and also if the model demonstrates high significance that is expressed in a value ranging between 0 and 1 (ideally it must be closer to 1) [25]. In this work, the R^2^ was 0.9207, 0.9197, 0.9499, and 0.9637 for each response (EE%, release, swelling, and sphericity respectively). Considering that the main goal of this experiment was to optimize, higher R^2^ values are important since they imply that the polynomial model is a very good predictor of the response. The higher the R^2^ values were, the better the polynomial was at either describing the system or making predictions about the system [26]. Additionally, the Adj R^2^ comprises the variation of the ordinary R^2^ and if the theoretical values adjust to the experimental data. In a practical point of view, if the Adj R^2^ is much lower than its R^2^, sample size might not be adequate to the model [27]. Regarding the obtained results for this parameter, all outputs present a reasonable Adj. R^2^. The Predicted R^2^ gives information concerning the suitability of the model in predicting new data. Regarding this, it is possible to observe that the model is more suitable to predict the swelling and less effective in the prediction of the release. The last evaluated parameter was the Adequate R^2^ that provides information about the signal to noise ratio. The obtained value must be higher than 4 to indicate an adequate signal. In Table 1 it is possible to observe that for all the outputs values higher than 4 were always achieved.

Through the ANOVA results present in Table S2 of the Supplementary Material it was possible to confirm that the model data were statistically significant for all the outputs since the probability value (*p*-value) was always lower than 0.05. Moreover, to understand if the model could correctly express data or, instead of it, if a more complex model should be applied, it was assessed the “lack of fit”. This parameter enables the comparison between the variability of the current model residuals and variability of the observations and replicative settings of the factors. The results obtained for all the outputs revealed that the *p*-value was not significant for the “Lack of fit”. In fact, a good valid model must present a significant value for its model (*p*-value < 0.05) and a non-significant value for its lack of fit (*p*-value > 0.05), thus suggesting that the model data is significant and it fits [28].

### 3.4. Model Validation

After the analysis of all responses provided by the DoE experiments, the optimal point was predicted taking into account the global aim of this study, which was to maximize the EE% and MPs sphericity while minimizing the protein release and MPs swelling. Regarding this information, the prediction of the ideal inputs was 4% of alginate and 6.6% of CaCl_2_. The desirability plot of the optimal point is presented in the Supplementary Material (Figure S1), and it is notorious that the combination of alginate concentration of 4% with calcium chloride concentration between 5.5 and 7.75% will allow us to reach the maximization and minimization previously defined for each output, since this graphical area presents a more warm color. Table 2 shows the confidence interval (C.I.) and the obtained results from the application of the optimal conditions in the microspheres production.

**Figure 3:**
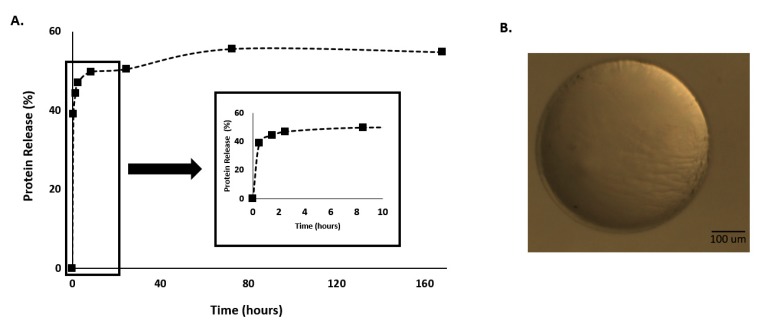
(**A**) Protein release profile and (**B**) morphological appearance of the Ca-Alginate microparticles (MPs) produced with the conditions defined by the predicted optimal point (4% of alginate and 6.6% of CaCl_2_).

The C.I. was applied to represent the range in which responses should lie 95% of the times. This means that: EE% must be between 76.11% and 90.75%; protein release should lie between 48.71% and 67.76%; MPs swelling must range between −18.31% and 3.39%; and finally MPs sphericity needs to be placed between 0.95 and 1. It is possible to observe from Table 2 that the obtained results were between the predicted C.I. proving that the used model had very good prediction behavior. Additionally, it is possible to say that using this model was possible to produce Ca-Alginate MPs with a large EE% since, from the initial amount of protein used, only 22% was not encapsulated in the produced system. Moreover, spherical MPs were also achieved, which could be observed through the coefficient of sphericity (it was obtained 0.97) very close to 1 (an example of a produced MP could be observed at Figure 3). Besides, all these results in combination with the swelling could be responsible for the promotion of the small protein release.

Therefore, Figure 2 presents a protein release profile obtained for the MPs formulated with the alginate and CaCl_2_ at the concentration defined in the predicted optimal point (4% of alginate and 6.6% of CaCl_2_).

Through Figure 3A, it is possible to observe a bi-phasic release with an initial burst effect during the first hours of the experiments. This initial burst release, which plays an important role in the therapeutic efficacy of MPs is commonly attributed to the release of drug located close to the surface of MPs. After a burst effect, the BSA is followed by a slower continuous release phase over the remaining experimental time [17].

Moreover, the swelling ability of hydrogels is a fundamental property that influences the drug release rates [29]. In fact, the ‘‘swelling–dissolution–erosion’’ process in alginate hydrogels is highly complex, with the osmotic pressure gradient between the gel and the environment playing an important role [17]. In this research work after 24 h the MPs produced with the conditions defined by the predicted optimal point present shrinking instead of swelling being a burst release of the protein presented during this period. This phenomenon could be explained by the proteins drug release mechanism from controlled delivery systems, which is often attributed to surface adhesion and desorption. The solubility of drugs as well as their partition coefficients affect the driving forces for release, and can lead to rapid release due to thermodynamic imbalances [30].

### 3.5. Influence of Other Divalent Ions in Cross-linked of Alginate MPs

Alginate MPs are usually produced by using Ca^2+^ as a gelling agent, under a process designed “ionotropic gelation”. However, these carriers can also be produced applying other divalent cations for the cross-linking step [31]. Moreover, the ability of these different divalent ions to perform gelation decreases as follow [6]:

Pb^2+^ > Cu^2+^ > Cd^2+^ > Ba^2+^ > Sr^2+^ > Ca^2+^ > Co^2+^, Ni^2+^, Zn^2+^ > Mn^2+^.

Regarding the above mentioned in this research work, we evaluated the influence of the divalent ions Cu^2+^, Ba^2+^, and Zn^2+^ (using the same conditions of the optimal point predicted by the DoE) in the selected outputs (EE%, release, swelling and sphericity; Table 3).

Through the obtained results, and against to what would be expected when an ion with higher crosslinking power is applied, it is possible to observe a decrease in release profile when cations with less affinity with alginate were used. This result could be related to the velocity of microsphere formation leading to an instantaneously high cross-linked shell and a core with few cations. Under these conditions, MPs have some resistance for the EE% but promote a quick release after the shell degradation. As an example, Vicini and collaborators (2017) [6] reported that Ba^2+^ ions promote an instantaneously high cross-linked shell around the microspheres, which difficult the further ions diffusion inside the spheres [6]. The same authors demonstrated that after 2 days in buffer the Ba-Alginate microspheres contained around 28% *w*/*w* of Ba^2+^ ions, at the adsorption plateau. In case of Ca-Alginate it was not promoted such highly crosslinked shell as what happened with Ba-Alginate particles leading to higher absorption of Ca^2+^ and to the achievement of the plateau value with of 43% *w*/*w* of Ca^2+^ [6]. The MPs sphericity does not present any standard behavior concerning the use of different divalent ions. Regarding this information, it can be important to clarify that the effect of the crosslinker type or affinity by the alginate natural polymer is not related to the effect of crosslinker concentration previously observed in the DoE study.

Moreover, and still considering the release output for the different alginate mixtures, it is interesting to visualize that Cu-alginate MPs presented the larger release and also a larger swelling effect after 24 h. Therefore, and as mentioned above, larger swelling rates are representative of a fast disintegration of the MPs, which lead to a fast release of the drug.

## 4. Conclusions

In the execution of this research work, a CCF model was applied from a mathematic DoE tool to quickly achieve the precise conditions to accomplish the production of Ca-alginate MPs through electrospraying, in order to achieve high EE%, a reduced protein release across the time and a homogeneous diffusion of the biomolecule delivered. Thus, the % of alginate and % of CaCl_2_ inputs was combined by DoE and a set of experiments was conducted in order to introduce the results of EE% and release, as well as MPs swelling and sphericity outputs, after 24 h. Among the obtained results, it was possible to highlight that the applied models presented to be statistically significant (*p*-value < 0.05), with a coefficient of determination of 0.9207, 0.9197, 0.9499, and 0.9637 for each output (EE%, release, swelling, and sphericity, respectively) and the optimal point was successfully validated. Overall, DoE demonstrated to be an interesting tool in the optimization of microparticulated systems, being possible to promote a panoply of combinations with the guarantee that in the end the best mathematical combination will be achieved.

## Figures and Tables

**Table 1:** Statistical coefficients.

Response	R^2^	Adjusted R^2^	Predicted R^2^	Adeq R^2^
EE%	0.9207	0.8413	0.3027	11.911
Release	0.9197	0.8394	0.3760	10.33
Swelling	0.9499	0.8998	0.7069	11.775
Sphericity	0.9637	0.9274	0.6514	12.679

**Table 2:** Confidence intervals and obtained results (*n* = 3) for the different responses evaluated.

Response	95% CI Low	95% CI High	Obtained Results
EE%	76.11	90.75	77.7 ± 2
Release	48.71	67.76	55 ± 9
Swelling	−18.39	3.39	−8.5 ± 2
Sphericity	0.95	1	0.97 ± 0.01

**Table 3:** Influence of several divalent ions on the alginate microspheres behavior and structure (*n* = 3).

Response	Cu^2+^	Ba^2+^	Ca^2+^
EE%	63.4	74.5	77.7
Release	81.8	52.5	55
Swelling	18.2	−3.97	−8.5
Sphericity	0.96	0.95	0.97

## Supplementary Materials

The following are available online at https://www.mdpi.com/2073-4360/11/12/1949/s1, Table S1: CCF design matrix and responses of the MPs in terms of EE%, release, swelling and sphericity; Table S2: ANOVA table of the CCF design model; Figure S1: Three-dimensional surface plot of the interaction of the different variables when the optimal conditions are selected.

## References

[1] Hernández R.M., Orive G., Murua A., Pedraz J.L. (2010). Microcapsules and microcarriers for in situ cell delivery. Adv. Drug Deliv. Rev..

[2] Banks S.R., Enck K., Wright M.W., Opara E.C., Welker M.E. (2019). Chemical Modification of Alginate for Controlled Oral Drug Delivery. J. Agric. Food Chem..

[3] Nagpal M., Maheshwari D., Rakha P., Dureja H., Goyal S., Dhingra G. (2012). Formulation development and evaluation of alginate microspheres of ibuprofen. J. Young Pharm..

[4] Deng X.-Q., Zhang H.-B., Wang G.-F., Xu D., Zhang W.-Y., Wang Q.-S., Cui Y.-L. (2019). Colon-specific microspheres loaded with puerarin reduce tumorigenesis and metastasis in colitis-associated colorectal cancer. Int. J. Pharm..

[5] Das M., Senapati P. (2008). Furosemide-loaded alginate microspheres prepared by ionic cross-linking technique: Morphology and release characteristics. Indian J. Pharm. Sci..

[6] Vicini S., Mauri M., Wichert J., Castellano M. (2017). Alginate gelling process: Use of bivalent ions rich microspheres. Polym. Eng. Sci..

[7] Partovinia A., Vatankhah E. (2019). Experimental investigation into size and sphericity of alginate micro-beads produced by electrospraying technique: Operational condition optimization. Carbohydr. Polym..

[8] Khorshidian N., Mahboubi A., Kalantari N., Hosseini H., Yousefi M., Arab M., Gomez da Cruz A. (2019). Chitosan-coated alginate microcapsules loaded with galactagogue herbs extract: Formulation optimization and characterization. Iran. J. Pharm. Res..

[9] Aderibigbe B.A., Buyana B. (2018). Alginate in wound dressings. Pharmaceutics.

[10] Cam M.E., Zhang Y., Edirisinghe M. (2019). Electrosprayed microparticles: A novel drug delivery method. Expert Opin. Drug Deliv..

[11] Szekalska M., Sosnowska K., Czajkowska-Kośnik A., Winnicka K. (2018). Calcium chloride modified alginate microparticles formulated by the spray drying process: A strategy to prolong the release of freely soluble drugs. Materials.

[12] Valente J., Sousa A., Queiroz J., Sousa F. (2019). DoE to improve supercoiled p53-pDNA purification by O-phospho-l-tyrosine chromatography. J. Chromatogr. B.

[13] Perucca Orfei C., Talò G., Viganò M., Perteghella S., Lugano G., Fabro Fontana F., Ragni E., Colombini A., De Luca P., Moretti M. (2018). Silk/Fibroin Microcarriers for Mesenchymal Stem Cell Delivery: Optimization of Cell Seeding by the Design of Experiment. Pharmaceutics.

[14] Sakai S., Ito S., Kawakami K. (2010). Calcium alginate microcapsules with spherical liquid cores templated by gelatin microparticles for mass production of multicellular spheroids. Acta Biomater..

[15] Voo W.-P., Lee B.-B., Idris A., Islam A., Tey B.-T., Chan E.-S. (2015). Production of ultra-high concentration calcium alginate beads with prolonged dissolution profile. RSC Adv..

[16] Valente J., Gaspar V., Antunes B.P., Countinho P., Correia I. (2013). Microencapsulated chitosan–dextran sulfate nanoparticles for controled delivery of bioactive molecules and cells in bone regeneration. Polymer.

[17] Patel N., Lalwani D., Gollmer S., Injeti E., Sari Y., Nesamony J. (2016). Development and evaluation of a calcium alginate based oral ceftriaxone sodium formulation. Prog. Biomater..

[18] Russo P., Zacco R., Rekkas D.M., Politis S., Garofalo E., Del Gaudio P., Aquino R.P. (2019). Application of experimental design for the development of soft-capsules through a prilling, inverse gelation process. J. Drug Deliv. Sci. Technol..

[19] Afonso A., Pereira P., Queiroz J.A., Sousa Â., Sousa F. (2014). Purification of pre-miR-29 by a new O-phospho-l-tyrosine affinity chromatographic strategy optimized using design of experiments. J. Chromatogr. A.

[20] Lotfipour F., Mirzaeei S., Maghsoodi M. (2012). Evaluation of the effect of CaCl2 and alginate concentrations and hardening time on the characteristics of Lactobacillus acidophilus loaded alginate beads using response surface analysis. Adv. Pharm. Bull..

[21] Shi P., He P., Teh T.K., Morsi Y.S., Goh J.C. (2011). Parametric analysis of shape changes of alginate beads. Powder Technol..

[22] Lee B.B., Ravindra P., Chan E.S. (2013). Size and shape of calcium alginate beads produced by extrusion dripping. Chem. Eng. Technol..

[23] Montgomery D.C. (2017). Design and Analysis of Experiments.

[24] Patil S.B., Sawant K.K. (2009). Development, optimization and in vitro evaluation of alginate mucoadhesive microspheres of carvedilol for nasal delivery. J. Microencapsul..

[25] Myers J.L., Well A.D., Lorch R.F. (2013). Research Design and Statistical Analysis.

[26] Balachandran M., Devanathan S., Muraleekrishnan R., Bhagawan S. (2012). Optimizing properties of nanoclay—Nitrile rubber (NBR) composites using face centred central composite design. Mater. Des..

[27] Haaland P.D. (1989). Experimental Design in Biotechnology.

[28] Gelman A. (2005). Analysis of variance—Why it is more important than ever. Ann. Stat..

[29] Carbinatto F.M., de Castro A.D., Evangelista R.C., Cury B.S. (2014). Insights into the swelling process and drug release mechanisms from cross-linked pectin/high amylose starch matrices. Asian J. Pharm. Sci..

[30] Huang X., Brazel C.S. (2001). On the importance and mechanisms of burst release in matrix-controlled drug delivery systems. J. Control. Release.

[31] Agulhon P., Robitzer M., Habas J.-P., Quignard F. (2014). Influence of both cation and alginate nature on the rheological behavior of transition metal alginate gels. Carbohydr. Polym..

