# Supplementary material for: Composite Central Face Design—An Approach to Achieve Efficient Alginate Microcarriers
Source: Polymers (Basel). 2019 Nov 27;11(12):1949. doi: 10.3390/polym11121949 (PMC6960800; doi:10.3390/polym11121949)
Supplement: Supplementary file 1 [file polymers-11-01949-s001.pdf]

Supplementary Materials

Composite Central Face Design—An Approach to Achieve Efficient Alginate Microcarriers

J.F.A Valente [1,*], J.R. Dias [1], A. Sousa [2] and N. Alves [1]

[1] CDRsp-IPL-Centre Rapid and Sustainable Product Development, Polytechnic Institute of Leiria, 2430-028 Marinha Grande, Portugal; juliana.dias@ipleiria.pt (J.R.D.); nuno.alves@ipleiria.pt (N.A.)

[2] CICS-UBI-Health Sciences Research Centre, Universidade da Beira Interior, Avenida Infante D. Henrique, 6200-506 Covilhã, Portugal; angela@fcsaude.ubi.pt

* Correspondence: joana.valente@ipleiria.pt; Tel.: +351 244 569 441

Table S1 – CCF design matrix and responses of the MPs in terms of protein entrapment, release, swelling and sphericity

Run	Alginate (%)	$CaCl_2$ (%)	Protein entrapment (%)	Release (after 24h)	Swelling (%)	Sphericity
1	4	10	74.4	70.8	-22	0.95
2	2.5	5.5	73.6	60.1	31	0.97
3	1	10	53	87.5	-4	0.84
4	2.5	5.5	76.7	67.1	22	0.98
5	1	5.5	59.2	90	-6	0.88
6	4	5.5	86	52	-8	0.97
7	4	1	76.9	67	4	0.96
8	2.5	1	71.3	73.4	23	0.94
9	1	1	65.4	95	-16	0.84
10	2.5	10	57.2	77.1	6	0.98
11	2.5	5.5	76.5	69	32	0.97

Table S2– ANOVA table of the CCF design model

Source	Sum of squares	Degrees of freedom	Mean square	F-value	P-value
**Protein entrapment**					
**Model**	934.85	5	186.97	11.61	0.0088
**A- Alginate (%)**	594.01	1	594.01	36.87	0.0017
**B- $CaCl_2$ (%)**	140.17	1	140.17	8.7	0.0319
**AB**	24.5	1	24.5	1.52	0.2723
**A^2**	0.16	1	0.16	9.828E-003	0.9249
**B^2**	166.21	1	166.21	10.32	0.0237
**Residual**	80.55	5	16.11		
**Lack of fit**	74.53	3	24.84	8.25	0.11
**Pure error**	6.02	2	3.01		
**Cor total**	1015.4	10			
**Release**					
**Model**	1559.78	5	311.95	11.45	0.0091
**A- Alginate (%)**	1139.88	1	1139.88	41.84	0.0013
**B- $CaCl_2$ (%)**	0.000	1	0.00	0.00	1
**AB**	31.92	1	31.92	1.17	0.3285
**A^2**	68.29	1	68.29	2.51	0.1742
**B^2**	225.86	1	225.86	8.29	0.0346
**Residual**	136.23	5	27.25		
**Lack of fit**	92.29	3	30.75	1.4	0.4424
**Pure error**	43.94	2	21.97		
**Cor total**	1696.01	10			
**Swelling**					
**Model**	3378.42	5	675.68	18.97	0.0029
**A- Alginate (%)**	0	1	0	0	1
**B- $CaCl_2$ (%)**	1160.17	1	160.17	4.5	0.0875
**AB**	361	1	361	10.13	0.0244
**A^2**	2185.01	1	2185.01	61.33	0.0005
**B^2**	156.84	1	156.84	4.4	0.09
**Residual**	178.12	5	35.62		
**Lack of fit**	117.46	3	39.15	1.29	0.4645
**Pure error**	60.67	2	30.33		
**Cor total**	3556.55	10			
**Sphericity**					
**Model**	0.029	5	5.719E003	26.58	0.0013
**A- Alginate (%)**	0.017	1	0.017	79.25	0.0003
**B- $CaCl_2$ (%)**	1.500E-0.004	1	1.500E-004	0.7	0.442
**AB**	2.500E-005	1	2.500E-005	0.12	0.7472
**A^2**	7.865E-00.3	1	7.885E-003	36.61	0.0018
**B^2**	1.095E-003	1	1.095E-003	5.08	0.0738
**Residual**	1.077E-003	5	2.154E-004		
**Lack of fit**	1.010E-003	3	3.367E-004	10.10	0.0914
**Pure error**	6.667E-005	2	3.333E-005		
**Cor total**	0.03	10			

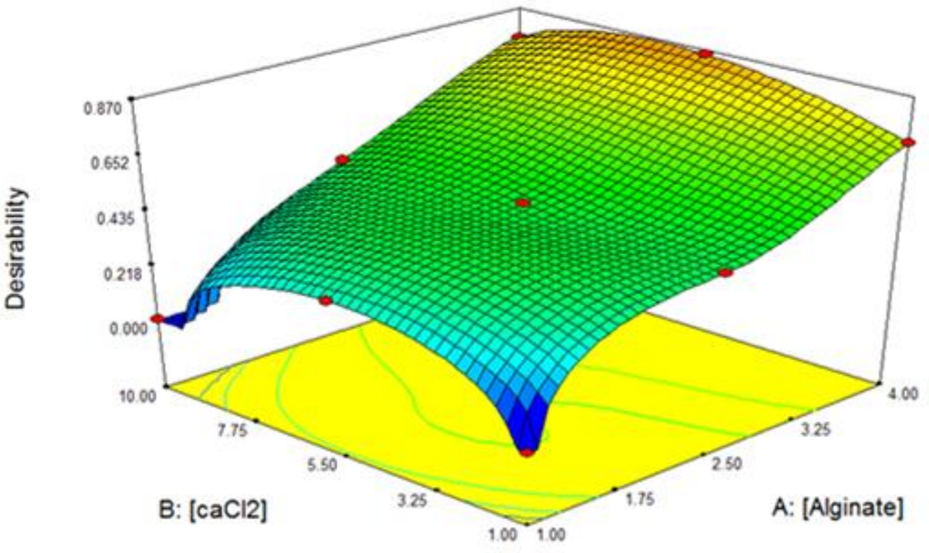

Figure S1– Three-dimensional surface plot of the interaction of the different variables when the optimal conditions are selected.
